# Dual regulation of miR-375 and CREM genes in pancreatic beta cells

**DOI:** 10.1080/19382014.2022.2060688

**Published:** 2022-04-04

**Authors:** David M. Keller, Isis G. Perez

**Affiliations:** Department of Biological Sciences, California State University Chico, Chico, CA, USA

**Keywords:** microRNA, miR-375, ββcells, CREM, transcription

## Abstract

MicroRNA-375 (miR-375) is upregulated in the islets of some diabetics and is correlated with poor outcome. Previous work in our laboratory showed that cyclic adenosine monophosphate (cAMP) reduces miR-375 expression and could provide a way to restore normal miR-375 levels, however the transcription repression mechanism is unknown. Using a chromatin immunoprecipitation assay we show that cAMP response element modulator (CREM) binds to the miR-375 promoter 3-fold above background and we find that CREM represses transcription from the miR-375 promoter 1.8-fold. While investigating miR-375 target genes we discovered that several microRNA:mRNA target prediction algorithms listed human CREM as a target gene of miR-375. The predicted binding site is conserved in primates but not in other species. We found that indeed miR-375 binds to the predicted site on human CREM and represses translation of a green fluorescent protein reporter gene by 30%. These findings suggest a primate-specific double-negative feedback loop, a mechanism that would keep these important β-cell regulators in check.

## Graphical Abstract

**Figure:**
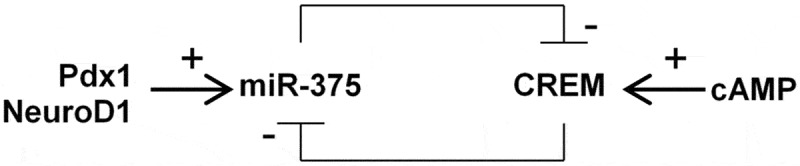

## Introduction

MicroRNA-375 (miR-375) is one of the most abundant miRNAs found in pancreatic islets, and is important for islet cell development and physiology.^1^ In zebrafish, a model for vertebrate development, targeted disruption of miR-375 by morpholinos severely disrupts islet development.^2^ In particular, miR-375 appears to control the ratio of β-cell to α-cells in the developing islets as miR-375 knockout mice have reduced numbers of β-cells and a corresponding increase in α-cells.^3^ In β-cell line cultures, miR-375 inhibits insulin secretion and β-cell replication, in part by inhibiting the protein synthesis of the mRNAs for myotrophin (Mtpn)^4^ and phosphoinositide 3-kinase-dependent-kinase (Pdk1).^5^

MiR-375 may play a role in the pathogenesis of type 2 diabetes mellitus in humans as well. In a small cohort of patients, Zhao et al.^6^ discovered that miR-375 expression in the pancreas was increased approximately 4-fold in diabetic patients compared with non-diabetic control individuals. While additional patient studies need to be done, it suggests that the miR-375 gene can be misregulated in the pathogenic state. Intriguingly, diabetic patients exhibit decreased β-cell mass and increased α-cell mass due in part to dedifferentiation of β-cells.^7^ In patients, the gain of miR-375 expression correlates with the decrease in β-cell to α-cell ratio.^6^ It is therefore at least a possibility that miR-375 contributes to the pathogenicity of diabetes by decreasing insulin secretion in β-cells,^4^ and by decreasing insulin levels through the reduction in β-cell numbers.^5,6^

We and others^5,8–11^ have made progress in the study of miR-375 gene regulation in order to determine its role in healthy and diabetic individuals. We initially identified the miR-375 promoter in independent genome-wide screens for binding sites for the transcription factors NeuroD1 and Pdx1.^10^ Avnit-Sagi et al.^8^ identified a 768 bp region upstream from miR-375 that directs its expression to pancreatic islets, and identified a TATA box and start site of transcription. Interestingly, they identified a 316 bp repression domain between the transcription start site and the miR-375 sequence. Work by El-Ouaamari et al.^5^ showed that elevated glucose could repress miR-375 expression, and we demonstrated that cAMP could repress as well.^11^ Therefore it is likely that the factor or factors responsible for repression will bind to the repression domain.

Cyclic-AMP-mediated transcriptional repression can occur via the protein CREM.^12^ CREM is in the CREB family of bZIP transcription factors and binds to the CRE sequence and is alternatively spliced. Depending upon the inclusion of a glutamine-rich activation domain, some CREM splice variants activate and some repress transcription.^13,14^ In addition, usage of an alternate intronic promoter generates an additional repressor called inducible-cAMP-elevated repressor (ICER),^15^ itself being activated by the cAMP – protein kinase A (PKA) axis. In β-cells there have been several repressing CREM and ICER splice variants identified^16,17^ which can repress gene transcription by recruiting histone deacetylase 1 to the promoter.^18^

We have proposed that one way in which cAMP enhances β-cell function over the long term is through repression of miR-375 through the cAMP – PKA pathway.^11^ In this model, cAMP agonists such as exendin-4 might enhance β-cell function in part through keeping miR-375 levels in check. To complete this model, however, it is essential to identify the factor or factors responsible for transcriptional repression of miR-375. Here we identify CREM as a regulator of miR-375 expression. Surprisingly, CREM mRNA itself is a target of miR-375, which suggests the presence of a double-negative feedback loop that keeps the expression of these two important regulators of β-cell function in check. Moreover, the CREM – miR-375 interaction is primate-specific and therefore may account for primate-specific aspects of β-cell regulation.

## Results

### INS-1 cells express CREM repressors only

**Figure 1.**
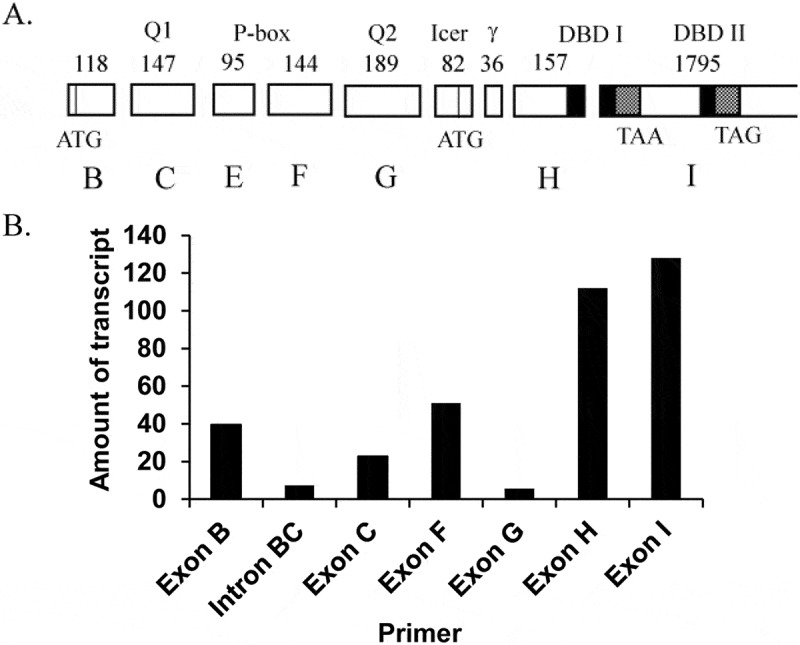
Identification of CREM exons that are expressed in INS-1 cells. (A) Schematic of the CREM locus. Functionality of each exon is shown on top, showing Q1 and Q2 (glutamine-rich transactivation domains), P-box (phosphorylation domain), γ (CREM-specific domain), and DBD I and II (DNA binding domains I and II). The length of each exon in bp is shown just above the exons. Exon labels B through I are shown below the exons. The CREM start codon is in exon B, while the ICER start codon is in the ICER exon. Either DBD I or II is spliced into the mature mRNA, with alternate stop codons as shown. The black shading represents the basic domains, while the gray shading represents the leucine zipper domains. (B) The transactivating exon G is not expressed in INS-1 cells. Total RNA was harvested from INS-1 cells and was used in reverse transcription reactions to generate cDNA. The cDNA was then used in quantitative real-time PCR using primers specific to the exon or intron denoted. Intron BC refers to the intron between exons B and C, and serves as a measure of background signal.

We initially suspected that CREM or ICER may repress the transcription of miR-375 because they are well-documented repressors activated by cAMP,^13^ and they control genes involved in β-cell function in the normal and pathogenic states.^16,19^ We wanted to perform a chromatin immunoprecipitation (ChIP) assay to test whether CREM was bound to the miR-375 promoter, but currently there are no antibodies that distinguish between the activating and repressing isoforms. We began by identifying the CREM exons that were expressed in rat insulinoma INS-1 832/13 cells (hereafter called INS-1) by using reverse transcription coupled with quantitative real-time PCR (qRT-PCR) (Figure 1(a)). Using exon-specific primers, we found that the majority of CREM exons were expressed in these cells (Figure 1(b)). A notable exception, however, was the critical activation exon G, the second of two glutamine-rich exons, and the key exon responsible for activation.^20^ The absence of this exon in CREM mRNA is strong evidence that there are only repressing isoforms present, thus any CREM protein detected by ChIP assay would be a repressing isoform.

Alt text: Panel A is a diagram of the CREM gene, showing 9 exons showing the location of the start codons, stop codons, and location of protein domains like the transcription activator domain and DNA binding domain. Panel B is a graph of reverse transcriptase reactions showing the exons that are transcribed into RNA in the INS-1 cells.

**Figure 2.**
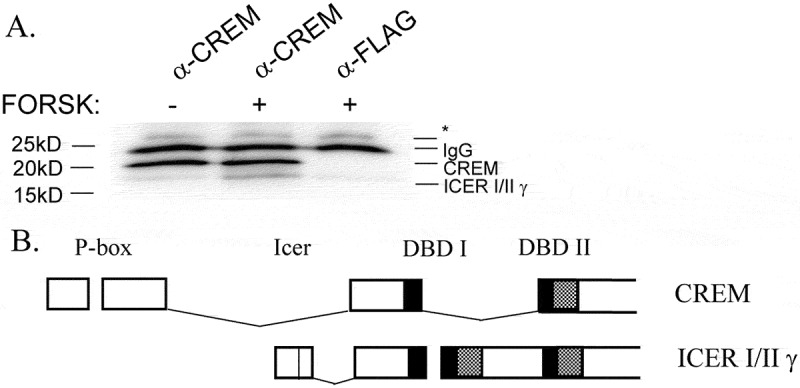
(A) CREM repressors expressed in β-cells. Immunoprecipitation-Western blot (IP-WB) detects two major CREM proteins at 20 kD and 18 kD which contain the C-terminal DNA binding domain. The 18 kD protein is likely ICERγ as it is induced by cAMP and migrates at the predicted size. The 20 kD protein is potentially CREMΦ2β. INS-1 cells were treated for 1 h with 10 μM forskolin (+) or DMSO control (-), then cells were harvested for IP-WB using monoclonal C-terminal anti-CREM 3B5 antibody or monoclonal anti-FLAG M5 antibody as a control. * indicates nonspecific proteins. (B) Potential isoform CREMΦ2β contains the regulatory phospho-domains and DNA binding domain II. ICER Iγ and IIγ are nearly identical sizes, and use either DNA binding domain I or II.

Through a combination of PCR and DNA sequencing using intron-spanning primers, we identified several transcript variants of CREM and ICER in INS-1 cells (data not shown). However, using an antibody against the essential DNA binding domain of CREM and ICER, we could only detect two isoforms that were translated into protein (Figure 2(a)). An 18 kD isoform was induced by forskolin treatment, and matches to the predicted size of either ICERIγ or IIγ (Figure 2(b)). A 20 kD isoform appears to be the most abundant isoform expressed, though its identity is unknown at this time (Figure 2(a)). Based on its predicted size and our DNA sequencing results (data not shown), it could be the rat ortholog of human CREMΦ2β, expressed from human CREM transcript variant 14. This variant contains the regulatory phosphorylation domains as well as DNA binding domain II (Figure 2(b)). In an attempt to identify the CREM isoforms expressed, we tested two additional antibodies, but neither could immunoprecipitate CREM (data not shown).

Alt text: Panel A is a picture of immunoprecipitation data showing protein expression of CREM and ICER. Panel B is two diagrams showing the exons that may be expressed based on evidence of protein expression in Panel A.

### CREM binds to the miR-375 promoter

**Figure 3.**
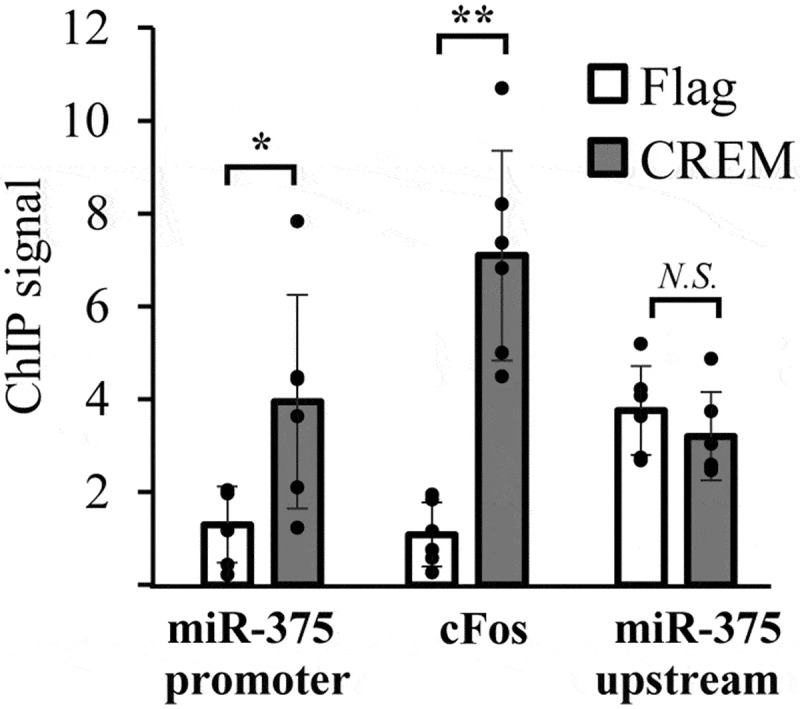
CREM binds to the miR-375 promoter in cells. ChIP assays were performed on INS-1 cells using anti-Flag and anti-CREM antibodies. Data represent qPCR results using primers that either amplify the proximal miR-375 promoter, the positive control cFos promoter, or a negative control sequence 2 kb distal to the miR-375 promoter. Bars represent averages, N = 6; * p < .05; ** p < .01. N.S. Not significant.

Having observed repressing CREM isoforms expressed in INS-1 cells, we next used the CREM antibody in a ChIP assay to determine if CREM binds to the miR-375 promoter sequence. As shown in Figure 3, CREM binding was enriched at the miR-375 promoter 3.0-fold compared to an anti-Flag antibody control (p = .046). In this assay, the c-Fos promoter was used as a positive control and a sequence 2 kb upstream from the miR-375 promoter was used as a negative control. Additional experiments showed that cAMP stimulation did not alter CREM occupancy on the promoter (data not shown). This is the situation for CREB, which can bind to promoters constitutively, but becomes active only upon cAMP stimulation,^21^ and argues against ICER being the repressor.

Alt text: A graph showing chromatin immunoprecipitation data.

**Figure 4.**
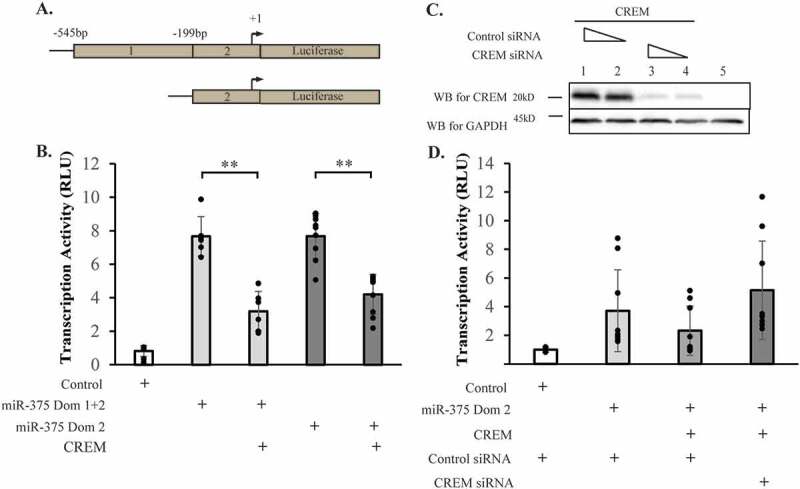
CREM represses miR-375 transcription via the conserved domain 2. (A) Schematic showing the miR-375 promoter conserved domains 1 and 2 linked to luciferase reporter genes. (B) Human embryonic kidney (HEK) 293 cells were transfected with luciferase genes attached to either a minimal promoter (Control) or the miR-375 promoter fragments, in conjunction with CREM. N = 9. (C) HEK 293 cells were transfected with CREM genes and with control or CREM small interfering RNA (siRNA). The expressed proteins were analyzed by Western blot using the CREM monoclonal antibody, and GAPDH expression was measured as a loading control. (D) Repression of the miR-375 luciferase construct is dependent on CREM, as reducing CREM relieves the transcriptional repression. N = 9. Bars represent averages and error bars represent ± 1 SD. * p < .05; ** p < .01; *** p < .001.

The proximal miR-375 promoter contains two conserved regions, labeled 1 and 2 in Figure 4(a). Both domains 1 and 2 or just domain 2 were used in luciferase reporter assays to localize the domain of CREM binding. Figure 4(b) shows that CREM binds to domain 2 and represses transcription 1.8-fold (p = 1.2 x 10^–5^). A small interfering RNA (siRNA) against CREM relieves this repression, demonstrating the specificity of the reaction (Figure 4(c-d)). HEK 293 T cells were used in transfection assays shown in the figure because they do not express CREM or miR-375, though the same results were observed in INS-1 cells (data not shown).

Alt text: Panel A is a diagram of the miR-375 promoter. Panel B is a graph of transcriptional activation data using a fusion of miR-375 promoter and a luciferase reporter gene. Panel C is a picture of a Western blot showing overexpression of CREM protein and knockdown of CREM protein by siRNA. Panel D is a graph of transcriptional activation data showing the effect of CREM siRNA on the transcription activity of the miR-375 promoter.

### miR-375 binds specifically to human CREM mRNA

**Figure 5.**
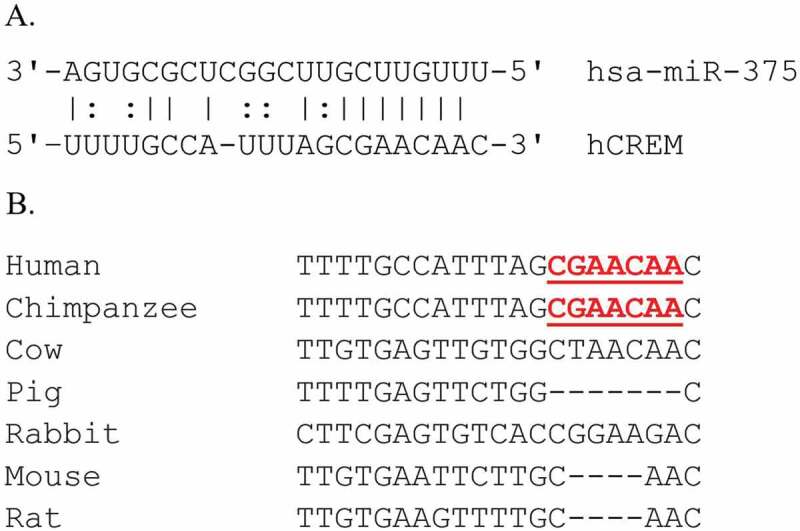
The human CREM mRNA is a predicted target of miR-375. (A) Predicted alignment between miR-375 and human CREM, revealing a perfect complementarity between a CREM 3ʹUTR sequence and the miR-375 seed sequence (nt 2–8). The CREM sequence spans human chromosome 10:35,212,743–35,212,763 bp (Genome Reference Consortium 38). G:U wobble base pairs are represented by the: symbol. (B) Alignment of CREM 3ʹUTR sequences between seven vertebrate species using T-Coffee, revealing perfect conservation between humans and chimpanzee, but not with rodents, at the miR-375 predicted binding site (underlined).

While researching potential targets of miR-375, we discovered that human CREM was a predicted target identified by several independent algorithms, including TargetScan,^22^ miRanda,^23^ and DIANA-microT-CDS^24,25^ (Figure 5(a)). However, no algorithm predicted rat or mouse CREM to be a miR-375 target gene, as rodent CREM lacks a complementary sequence to miR-375ʹs seed region (Figure 5(b)). For example, TargetScan identified an exact match between human CREM and nucleotides 2–8 of hsa-miR-375 (7mer-m8 site), but classified the interaction as poorly conserved.^22^

Alt text: Panel A shows complementary base pairing between miR-375 and a 3ʹUTR sequence in the human CREM gene. Panel B is a multiple sequence alignment of the possible miR-375 binding site in the CREM gene between several mammal species.

**Figure 6.**
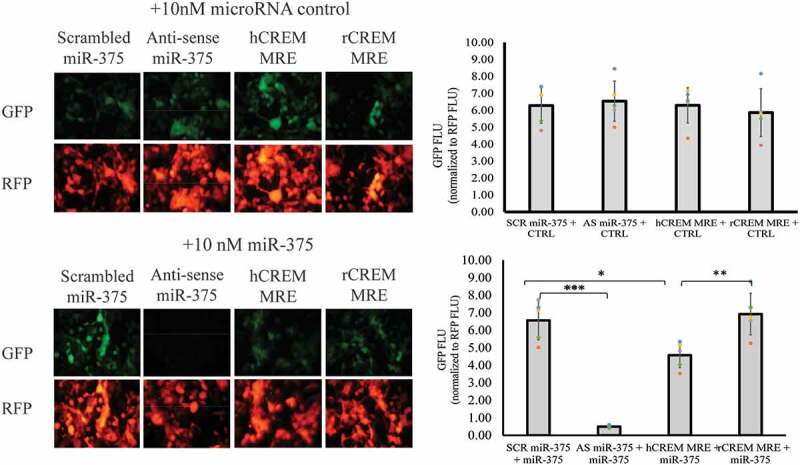
miR-375 binds to the predicted human CREM binding site. HEK 293 T cells were transfected with GFP reporter genes fused with either a negative control scrambled miR-375 sequence (SCR miR-375), positive control anti-sense miR-375 (AS miR-375), human CREM miRNA recognition element (hCREM MRE), or rat CREM sequence (rCREM MRE). (A) Cells were co-transfected with either 10 nM control *C. elegans* miRNA (CTRL) or (B) 10 nM miR-375. Finally, RFP was co-transfected to normalize for cell density and transfection efficiency. Cells were imaged by fluorescent microscopy and graphs were generated by plate reader fluorometer (FLU = fluorescence light units). N = 5. Bars represent averages and error bars represent ± 1 SD. * p < .05; ** p < .01; *** p < .001.

To test whether miR-375 might regulate human CREM specifically, we cloned the miR-375 miRNA recognition element (MRE) from human CREM (hCREM) at the 3’ end of the green fluorescent protein (GFP) gene. We also cloned the homologous sequence from rat CREM (rCREM), although the miR-375 binding site is not conserved. Our positive control was a perfectly complementary match to miR-375 (anti-sense, AS-mir375) and negative control was a scrambled sequence (SCR-mir375) predicted not to be regulated by any miRNA in β-cells. We co-transfected either miR-375 or a *C. elegans* miRNA control predicted to not target any mammalian mRNA. We used HEK 293 T cells because they lack endogenous miR-375. Results showed that the miRNA control sequence did not bind to any of our GFP reporter genes (Figure 6(a)), while miR-375 bound specifically to the hCREM MRE and repressed GFP expression by 30% (p = .0138) compared to the rCREM sequence (Figure 6(b), compare columns 3 and 4). This supports the miRNA target prediction algorithm results that the miR-375:CREM interaction is human-specific.

Alt text: In total, 16 fluorescent pictures separated into four rows of four pictures each. In rows one and three the cells are visualized for GFP fluorescence and rows two and four are visualized for RFP fluorescence. On the right side of the figure, there are two graphs which were derived from the fluorescent pictures.

## Discussion

Previous work in our laboratory demonstrated that miR-375 is transcriptionally repressed by cAMP signaling through PKA.^11^ Here we build on that previous work by showing that the cAMP-regulated transcription repressor CREM binds to the miR-375 promoter (Figure 3) and represses transcription (Figure 4(b,d)). While no cAMP response element was identified in the miR-375 promoter, there is a conserved AP1 site, 5’-TGAGTCA-3’, in domain 2 of the miR-375 promoter which may provide a binding site for CREM.^26–28^ Using miRNA target prediction algorithms we found that CREM is a target gene of miR-375, but specifically in humans and other primates (Figure 5) and subsequently showed that miR-375 can bind specifically to the human CREM MRE (Figure 6). Consistent with our results for miR-375, several studies in β-cells have shown that other genes are down-regulated in response to cAMP.^16,19,29^ In one study, several genes necessary for insulin secretion were repressed by hyperglycemic conditions in a PKA-dependent manner.^19^ It was hypothesized that this mechanism contributed to β-cell failure in type 2 diabetes.

**Figure 7.**
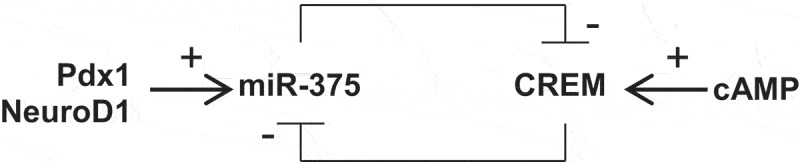
Model for miR-375 and CREM regulation in human β-cells. Hypothesis for a double-negative feedback loop in human β-cells, which predicts that two bistable states exist: miR-375 ‘on’ or CREM ‘on,’ depending upon the relative strength of the activating inputs.

Reciprocal regulation by miRNAs and transcriptional repressors is a recurrent theme in mammalian cells.^30^ These double-negative feedback loops play a variety of roles in cells, for example by reinforcing cell fate decisions,^31,32^ by synchronizing biological oscillators,^33,34^ by dampening protein fluctuations,^35^ and by increasing transcriptional response times.^36^ As shown in Figure 7, our model for a double-negative feedback loop predicts that miR-375 is activated by factors such as Pdx1 and NeuroD1,^10^ and then it subsequently represses CREM gene expression. If CREM activity is triggered by cAMP signaling, then miR-375 would be accordingly repressed. This double-negative feedback loop may switch the system between two states, either miR-375 ‘on’ or CREM ‘on,’ depending on the relative strength of the activating inputs. This may have important consequences for β-cells, as CREM isoforms have been shown to repress genes involved in insulin production and secretion.^17,19^

Alt text: A diagram using arrows and block arrows for how miR-375 and CREM may alternately repress each other’s expression.

Intriguingly, the miR-375 MRE is present only in the CREM transcript of primates, suggesting a fundamental difference in CREM regulation in primates compared with other species. Due to the sequence conservation of the miR-375 promoter in primates and rodents, we hypothesize that CREM represses miR-375 transcription in a cAMP-dependent fashion in both, but that the feedback loop has evolved only in the primate lineage. This finding supports the model that miRNA tend to be more evolutionarily conserved than their target sequences.^37^ Indeed, miR-375 is conserved perfectly in all mammals analyzed, yet the CREM MRE is perfectly conserved only in primates. Thus our study, along with others,^38,39^ highlights a limitation of using rodents in β-cell research.

## Materials and methods

### Cell culture

Rat insulinoma INS-1 832/13 cells were a gift from Dr. Chris Newgard (Duke University Medical Center). Cells were grown in RPMI-1640 media (Corning #10041CV) containing 10% heat-inactivated fetal bovine serum (FBS, Gibco #10437028), 100 units/ml penicillin and streptomycin (Hyclone #SV30082.01), 1 mM sodium pyruvate (Hyclone #SH30239.01), and 50 μM 2-mercaptoethanol (Sigma-Aldrich #M6250). Human embryonic kidney 293 T cells (HEK-293 T, American Type Culture Collection #CRL-3216) were grown in DMEM (Corning #10017CV) containing 10% FBS and penicillin and streptomycin. Cells were grown at 37°C in a humidified chamber containing 5% CO_2_.

### Reverse transcription

RNA was extracted from INS-1 832/13 cells with TRIzol (Invitrogen #15596026) and 500 ng RNA was used for first-strand cDNA synthesis in a reaction containing 50 ng of random primers (Promega #C1181), 500 μM dNTPs (Fisher Bioreagents #BP2564-1), 1× first-strand buffer, and 200 units of Moloney murine leukemia virus reverse transcriptase (Promega #M1705).

### Antibodies

1 μg CREM monoclonal 3B5 antibody (Abnova #H00001390M02) or Flag monoclonal antibody (Agilent Technologies #200474) were used for immunoprecipitation and chromatin immunoprecipitation (ChIP). Western blots were conducted with either 1:500 dilutions of the above antibodies or 1:10,000 dilution of a rabbit monoclonal GAPDH antibody (Abcam #AB181602).

### Chromatin Immunoprecipitation (ChIP)

INS-1 832/13 cells were fixed with 1% paraformaldehyde in 1x PBS for 15 min at room temperature (RT) and the cross-linking reaction was stopped with the addition of 125 mM glycine for 5 min. The cells were washed with 1x PBS on ice and harvested by cell scraping in 1x PBS. Cells were centrifuged at 1000x g at 4°C for 5 min and cell pellets were lysed in 600 μl buffer containing 20 mM Tris-HCl pH 8.1, 150 mM NaCl, 0.1% SDS, and 0.5% Triton X-100. Chromatin was sonicated to an average size of 2 kb using a Vibra-Cell probe sonicator (5 x 15 sec) (Sonics & Materials, Inc.). Samples were centrifuged and supernatants were pre-cleared for 1 h at 4°C with protein G agarose blocked with salmon sperm DNA (MilliporeSigma #16201). 500 μg of supernatant was rotated overnight at 4°C with CREM or Flag antibodies. Following antibody incubation, 50 μl protein G agarose (50% slurry) was added for 2 h at 4°C, then sequentially washed with lysis buffer 2 times for 10 min each, LiCl buffer (10 mM Tris-HCl pH 8.1, 250 mM LiCl, 1 mM EDTA, 1% NP-40, 1% sodium deoxycholate) once for 10 min, and 2 times with TE buffer (50 mM Tris-HCl pH 8.1, 1 mM EDTA). Elution buffer (100 mM NaHCO3 and 1% SDS) was added directly to the beads and the immuno-complexes were dissociated in two sequential washes of 200 μl each for 15 min at RT. The supernatants were pooled and incubated at 65°C overnight to reverse the formaldehyde crosslinking. The samples were extracted with 25:24:1 phenol:chloroform:isoamyl alcohol and nucleic acids were precipitated with ethanol. Each ChIP sample was resuspended in 100 μl 10 mM Tris-HCl pH 8.0. Data was plotted as ChIP signal in which quantitative PCR products were quantitated by comparison to a ChIP input standard curve.

### Quantitative PCR

ChIP DNA or cDNA (3 ul) was analyzed in a 15 μl real-time PCR (qPCR) containing 1× SYBR green mix (Thermo Scientific #K0381) and 0.25 μM primer pairs. Reactions were run in a Realplex 2 (Eppendorf) for 15 min at 95°C, followed by 40 cycles of 15 sec at 95°C and 45 sec at 68°C. ChIP primers included miR-375 upstream (5’-TCCTATCCCTGCCCTCCAGCTTT-3’ and 5’-CTTCACCATCCTCTTGCCCTGCT-3’), miR-375 promoter primers (5’- GCCAATTCAGTCTCTCGCCCCTA-3’ and 5’- CCCCGGACAGGTGTGTGTGTG-3’) and cFos promoter (5’- CCTCCAGTTTCTCTGTTCCGCTCA-3’ and 5’- CGGCTCTATCCAGTCTTCTCAGTTGC-3’).

### Luciferase assay

HEK 293 T cells were transfected using lipofectamine 2000 (Invitrogen #11668027) with 50 ng plasmids expressing firefly luciferase, including pGL3-Basic (Promega #E1751) and pGL3-Basic + rat miR-375 promoter fragments. Promoter conserved domains 1 and 2 span base pair positions +10 to – 545, while domain 2 spans positions +10 to −199. pSV-CREMα was a gift from Paolo Sassone-Corsi^13^ and mouse CREM small interfering RNA (siRNA) sequence was 5’-GCCTGCACAGTCCCCAGCA-3’ (Ambion). After 48 h luciferase assays were conducted using the Promega Dual-Glo Luciferase Assay System (#E2920) and samples were analyzed in a BioTek Synergy H1 multi-mode plate reader. Data was plotted as Transcriptional activity in relative light units (RLU).

### MicroRNA reporter assay

HEK 293 T cells were transfected with plasmids derived from pEGFP-C1 (Clontech #6084-1). Reporter sequences were cloned at the 3’ end of the green fluorescent protein (GFP) gene, following an added stop codon and rv primer 4 site for sequencing purposes. MiR-375 anti-sense sequence is 5’- TCACGCGAGCCGAACGAACAAA-3’, scrambled sequence is 5’- AAGAGCGGCGAACCACTACACA-3’, human CREM microRNA recognition element (MRE) sequence is 5’- TTTTGCCATTTAGCGAACAAC-3’, and the homologous rat CREM sequence is 5’- TCCATTGTGAAGTTTTGCAAC-3’. mRFP-N1 plasmid expressing the red fluorescent protein gene was used as an internal control (Addgene #54635). Cells were co-transfected with 10 nM miRIDIAN miR-375 mimic (Dharmacon #C-300682-05) or negative control #1 based on cel-miR-67 (Dharmacon # CN-001000-01). 48 h post transfection cells were imaged with an Olympus CKX41 inverted microscope followed by lysis and analysis in a BioTek Synergy H1 multi-mode plate reader. Data was plotted as GFP fluorescence light units (FLU) normalized to RFP FLU.

### Statistics

Samples were analyzed by two-tailed, paired Student t-tests. Averages were plotted in Microsoft Excel with error bars representing ± 1 standard deviation.

## Figures and Tables

**Table:** 

AP1	activator protein 1
AS	anti-sense
bZIP	basic leucine zipper
ChIP	chromatin immunoprecipitation
cAMP	cyclic adenosine monophosphate
CRE	cAMP response element
CREM	CRE modulator
hCREM	human CREM
rCREM	rat CREM
CREB	CRE binding protein
GFP	green fluorescent protein
HEK 293T	human embryonic kidney 293T
ICER	inducible-cAMP-elevated repressor;
MRE	microRNA recognition element
PKA	protein kinase A
qRT-PCR	reverse transcription quantitative real-time polymerase chain reaction
siRNA	small interfering RNA
SCR	scrambled

## References

[1] Li X. MiR-375, a microRNA related to diabetes. Gene. 2014;533(1):1–4. doi:10.1016/j.gene.2013.09.105. 24120394

[2] Kloosterman WP, Lagendijk AK, Ketting RF, Moulton JD, Plasterk RHA, Carrington JC. Targeted inhibition of miRNA maturation with morpholinos reveals a role for miR-375 in pancreatic islet development. PLoS Biol. 2007;5(8):e203. doi:10.1371/journal.pbio.0050203. 17676975 PMC1925136

[3] Poy MN, Hausser J, Trajkovski M, Braun M, Collins S, Rorsman P, Zavolan M, Stoffel M. miR-375 maintains normal pancreatic α- and β-cell mass. Proc Natl Acad Sci U S A. 2009;106(14):5813–5818. doi:10.1073/pnas.0810550106. 19289822 PMC2656556

[4] Poy MN, Eliasson L, Krutzfeldt J, Kuwajima S, Ma X, MacDonald PE, Pfeffer S, Tuschl T, Rajewsky N, Rorsman P, et al. A pancreatic islet-specific microRNA regulates insulin secretion. Nature. 2004;432(7014):226–230. doi:10.1038/nature03076. 15538371

[5] El Ouaamari A, Baroukh N, Martens GA, Lebrun P, Pipeleers D, van Obberghen E. miR-375 targets 3’-phosphoinositide-dependent protein kinase-1 and regulates glucose-induced biological responses in pancreatic beta-cells. Diabetes. 2008;57(10):2708–2717. doi:10.2337/db07-1614. 18591395 PMC2551681

[6] Zhao H, Guan J, Lee H-M, Sui Y, He L, Siu JJ, Tse PPP, Tong PCY, Lai FMM, Chan JCN, et al. Up-regulated pancreatic tissue microRNA-375 associates with human type 2 diabetes through beta-cell deficit and islet amyloid deposition. Pancreas. 2010;39(6):843–846. doi:10.1097/MPA.0b013e3181d12613. 20467341

[7] Talchai C, Xuan S, Lin HV, Sussel L, Accili D. Pancreatic β cell dedifferentiation as a mechanism of diabetic β cell failure. Cell. 2012;150(6):1223–1234. doi:10.1016/j.cell.2012.07.029. 22980982 PMC3445031

[8] Avnit-Sagi T, Kantorovich L, Kredo-Russo S, Hornstein E, Walker MD, Maedler K. The promoter of the pri-miR-375 gene directs expression selectively to the endocrine pancreas. PLoS One. 2009;4(4):e5033. doi:10.1371/journal.pone.0005033. 19343226 PMC2660411

[9] Avnit-Sagi T, Vana T, Walker MD. Transcriptional mechanisms controlling miR-375 gene expression in the pancreas. Exp Diabetes Res. 2012;2012:891216. doi:10.1155/2012/891216. 22778717 PMC3388352

[10] Keller DM, McWeeney S, Arsenlis A, Drouin J, Wright CVE, Wang H, Wollheim CB, White P, Kaestner KH, Goodman RH, et al. Characterization of Pancreatic Transcription Factor Pdx-1 Binding Sites Using Promoter Microarray and Serial Analysis of Chromatin Occupancy. J Biol Chem. 2007;282(44):32084–32092. doi:10.1074/jbc.M700899200. 17761679

[11] Keller DM, Clark EA, Goodman RH. Regulation of microRNA-375 by cAMP in pancreatic β-cells. Mol Endocrinol. 2012;26(6):989–999. doi:10.1210/me.2011-1205. 22539037 PMC3355542

[12] Rosenberg D, Groussin L, Jullian E, Perlemoine K, Bertagna X, Bertherat J. Role of the PKA-regulated transcription factor CREB in development and tumorigenesis of endocrine tissues. Ann N Y Acad Sci. 2002;968(1):65–74. doi:10.1111/j.1749-6632.2002.tb04327.x. 12119268

[13] Foulkes NS, Borrelli E, Sassone-Corsi P. CREM gene: use of alternative DNA-binding domains generates multiple antagonists of cAMP-induced transcription. Cell. 1991;64(4):739–749. doi:10.1016/0092-8674(91)90503-q. 1847666

[14] Delmas V, Laoide BM, Masquilier D, de Groot RP, Foulkes NS, Sassone-Corsi P. Alternative usage of initiation codons in mRNA encoding the cAMP-responsive-element modulator generates regulators with opposite functions. Proceedings of the National Academy of Sciences, USA. 1992;89:4226–4230. doi: 10.1073/pnas.89.10.4226. PMC49054 1584756

[15] Molina CA, Foulkes NS, Lalli E, Sassone-Corsi P. Inducibility and negative autoregulation of CREM: an alternative promoter directs the expression of ICER, an early response repressor. Cell. 1993;75(5):875–886. doi:10.1016/0092-8674(93)90532-u. 8252624

[16] Inada A, Yamada Y, Someya Y, Kubota A, Yasuda K, Ihara Y, Kagimoto S, Kuroe A, Tsuda K, Seino Y, et al. Transcriptional repressors are increased in pancreatic islets of type 2 diabetic rats. Biochem Biophys Res Commun. 1998;253(3):712–718. doi:10.1006/bbrc.1998.9833. 9918792

[17] Inada A, Someya Y, Yamada Y, Ihara Y, Kubota A, Ban N, Watanabe R, Tsuda K, Seino Y. The cyclic AMP response element modulator family regulates the insulin gene transcription by interacting with transcription factor IID. J Biol Chem. 1999;274(30):21095–21103. doi:10.1074/jbc.274.30.21095. 10409662

[18] Tenbrock K, Juang Y-T, Leukert N, Roth J, Tsokos GC. The transcriptional repressor cAMP response element modulator alpha interacts with histone deacetylase 1 to repress promoter activity. J Immunol. 2006;177(9):6159–6164. doi:10.4049/jimmunol.177.9.6159. 17056544

[19] Abderrahmani A, Cheviet S, Ferdaoussi M, Coppola T, Waeber G, Regazzi R. ICER induced by hyperglycemia represses the expression of genes essential for insulin exocytosis. EMBO J. 2006;25(5):977–986. doi:10.1038/sj.emboj.7601008. 16498408 PMC1409716

[20] Laoide BM, Foulkes NS, Schlotter F, Sassone-Corsi P. The functional versatility of CREM is determined by its modular structure. EMBO J. 1993;12(3):1179–1191. doi:10.1002/j.1460-2075.1993.tb05759.x. 8458330 PMC413321

[21] Chrivia JC, Kwok RP, Lamb N, Hagiwara M, Montminy MR, Goodman RH. Phosphorylated CREB binds specifically to the nuclear protein CBP. Nature. 1993;365(6449):855–859. doi:10.1038/365855a0. 8413673

[22] Friedman RC, Farh KK-H, Burge CB, Bartel DP. Most mammalian mRNAs are conserved targets of microRNAs. Genome Res. 2008;19(1):92–105. doi:10.1101/gr.082701.108. 18955434 PMC2612969

[23] Enright AJ, John B, Gaul U, Tuschl T, Sander C, Marks DS. MicroRNA targets in Drosophila. Genome Biol. 2003;5(1):R1. doi:10.1186/gb-2003-5-1-r1. 14709173 PMC395733

[24] Paraskevopoulou MD, Georgakilas G, Kostoulas N, Vlachos IS, Vergoulis T, Reczko M, Filippidis C, Dalamagas T, Hatzigeorgiou AG. DIANA-microT web server v5.0: service integration into miRNA functional analysis workflows. Nucleic Acids Res. 2013;41(W1):W169–173. doi:10.1093/nar/gkt393. 23680784 PMC3692048

[25] Reczko M, Maragkakis M, Alexiou P, Grosse I, Hatzigeorgiou AG. Functional microRNA targets in protein coding sequences. Bioinformatics. 2012;28(6):771–776. doi:10.1093/bioinformatics/bts043. 22285563

[26] Macián F, López-Rodríguez C, Rao A. Partners in transcription: NFAT and AP-1. Oncogene. 2001;20(19):2476–2489. doi:10.1038/sj.onc.1204386. 11402342

[27] Bodor J, Habener JF. Role of Transcriptional Repressor ICER in Cyclic AMP-mediated Attenuation of Cytokine Gene Expression in Human Thymocytes. J Biol Chem. 1998;273(16):9544–9551. doi:10.1074/jbc.273.16.9544. 9545284

[28] Rutberg SE, Adams TL, Olive M, Alexander N, Vinson C, Yuspa SH. CRE DNA binding proteins bind to the AP-1 target sequence and suppress AP-1 transcriptional activity in mouse keratinocytes. Oncogene. 1999;18:1569–1579. doi:10.1038/sj.onc.1202463. 10102627

[29] Zhou Y-P, Marlen K, Palma JF, Schweitzer, A, Reilly, L, Gregoire, FM, Xu, GG, Blume, JE, Johnson, JD. Overexpression of repressive cAMP response element modulators in high glucose and fatty acid-treated rat islets. A common mechanism for glucose toxicity and lipotoxicity? J Biol Chem. 2003;278(51):51316–51323. doi:10.1074/jbc.M307972200. 14534319

[30] Tsang J, Zhu J, van Oudenaarden A. MicroRNA-mediated feedback and feedforward loops are recurrent network motifs in mammals. Mol Cell. 2007;26(5):753–767. doi:10.1016/j.molcel.2007.05.018. 17560377 PMC2072999

[31] Johnston RJ, Chang S, Etchberger JF, Ortiz CO, Hobert O. MicroRNAs acting in a double-negative feedback loop to control a neuronal cell fate decision. Proc Natl Acad Sci U S A. 2005;102(35):12449–12454. doi:10.1073/pnas.0505530102. 16099833 PMC1194938

[32] Li X, Carthew RW. A microRNA mediates EGF receptor signaling and promotes photoreceptor differentiation in the Drosophila eye. Cell. 2005;123(7):1267–1277. doi:10.1016/j.cell.2005.10.040. 16377567

[33] Bonev B, Stanley P, Papalopulu N. MicroRNA-9 Modulates Hes1 ultradian oscillations by forming a double-negative feedback loop. Cell Rep. 2012;2(1):10–18. doi:10.1016/j.celrep.2012.05.017. 22840391 PMC4103481

[34] Kim J-R, Shin D, Jung SH, Heslop-Harrison P, Cho K-H. A design principle underlying the synchronization of oscillations in cellular systems. J Cell Sci. 2010;123(4):537–543. doi:10.1242/jcs.060061. 20103537

[35] Becskei A, Serrano L. Engineering stability in gene networks by autoregulation. Nature. 2000;405(6786):590–593. doi:10.1038/35014651. 10850721

[36] Rosenfeld N, Elowitz MB, Alon U. Negative autoregulation speeds the response times of transcription networks. J Mol Biol. 2002;323(5):785–793. doi:10.1016/s0022-2836(02)00994-4. 12417193

[37] Barbash S, Shifman S, Soreq H. Global Coevolution of Human MicroRNAs and Their Target Genes. Mol Biol Evol. 2014;31(5):1237–1247. doi:10.1093/molbev/msu090. 24600049

[38] Li J, Liu Y, Xin X, Kim TS, Cabeza EA, Ren J, Nielsen R, Wrana JL, Zhang Z. Evidence for Positive Selection on a Number of MicroRNA Regulatory Interactions during Recent Human Evolution. PLoS Genet. 2012;8(3):e1002578. doi:10.1371/journal.pgen.1002578. 22457636 PMC3310733

[39] McLoughlin HS, Wan J, Spengler RM, Xing Y, Davidson BL. Human-specific microRNA regulation of FOXO1: implications for microRNA recognition element evolution. Hum Mol Genet. 2014;23(10):2593–2603. doi:10.1093/hmg/ddt655. 24368418

